# Making variation visible

**DOI:** 10.1007/s12471-026-02027-9

**Published:** 2026-02-16

**Authors:** Pim van der Harst

**Affiliations:** Department of Cardiology, University Medical Centre Groningen, Groningen, The Netherlands

This issue features four interesting papers, each addressing a familiar clinical dilemma where our usual approach does not clearly point to the best next step.

Magni et al. study repeat ablation for atrial fibrillation in patients with durably isolated pulmonary veins [1]. Operators add extra lesions, posterior wall ablation, in registry, no repeat strategy improved arrhythmia-free survival after adjustment. Markers of atrial disease, left atrial size, were more informative for recurrence risk. The key message is that adding lesions at the second procedure does not reliably improve outcomes; studies tailoring strategy to atrial phenotype rather than operator preference are needed.

Two papers focus on mitral regurgitation (MR). El Mathari et al. compare early mitral valve repair with active surveillance (with surgery once guideline triggers develop) in asymptomatic severe primary MR [2]. About half of the surveilled patients ultimately required surgery, with comparable postoperative outcomes between early and facilitated surgery. This supports structured surveillance while highlighting the challenge of identifying, at baseline, which ‘asymptomatic’ patients might benefit from being prioritised.

Carvalheiro et al. examine secondary MR and show that prognosis involves more than MR grade. Left atrial reservoir strain predicted mortality, and pairing it with tricuspid regurgitation velocity enhanced risk stratification. Clinically, this highlights relevance of assessing atrial function and right-sided load [3].

Finally, Drost et al. take a health-system view [4]. They report substantial regional differences in LVAD utilisation across the Netherlands over 2015–2024, without clear variation across socioeconomic categories. Even without an SES signal, variation matters: it reflects referral patterns, access, and timing of recognition—factors we can measure, benchmark, and improve upon.

Together, these studies point in the same direction: improved phenotyping and clearer pathways, so variation in care becomes something we understand and can improve upon.
